# Change in Age of Diagnosis and Demographics of Type 1 Diabetes Mellitus During the COVID-19 Era

**DOI:** 10.1155/pedi/7276579

**Published:** 2025-10-16

**Authors:** Katya Sracic, Naveen Uli, Ryan Heksch

**Affiliations:** ^1^Department of Medical Education, Akron Children's Hospital, Akron, Ohio, USA; ^2^Division of Endocrinology, Cincinnati Children's Hospital, Cincinnati, Ohio, USA; ^3^Center for Diabetes and Endocrinology, Akron Children's Hospital, Akron, Ohio, USA

## Abstract

Since COVID-19 onset, pediatric endocrinologists have been making an assumption that there was a shift in diagnosis age of type 1 diabetes mellitus (T1DM) to younger children. Younger children are more likely to present in DKA, are more difficult to diagnose and treat, and age at diagnosis can affect prognosis. We performed a retrospective chart review of patients diagnosed with T1DM for 3 years before COVID-19 and the 3 years during COVID-19. Demographics were evaluated using the Chi-squared test for categorical data and Student's *t*-test or ANOVA for continuous data. During this time, 698 patients were diagnosed with T1DM, with more patients during COVID-19. The average age of diagnosis significantly increased by 0.7 years (*p*=0.025). There was a significant difference in the distribution of age groups between the two time periods (*p*=0.0065). There was a significant decrease in new cases among patients between the ages of 2–5 years from 2017 to 2020, a transient finding as they reverted back to previous rates by 2022. New diagnoses between 13 and 18 years were increasing prior to 2020 (7%–23%), subsequently leveling out. Patients were 1.6 times more likely to present in DKA during COVID-19; however, there was no significant change in hemoglobin A1c (HbA1c). There was no significant change in thyroperoxidase (TPO) antibody positivity. There was a significant decrease (*p*=0.018) in patients with elevated tissue transglutaminase (TTG)-IgA from pre-COVID to post-COVID. Average age at diagnosis in our cohort increased since the start of COVID-19, contradicting previous studies and our hypothesis. The number of new cases increased, and the age distribution changed. There was a significant decrease in number of younger patients in 2020, followed by a normalization of new cases in those 2–5 years old, which may have led to the belief that more toddlers were being diagnosed. The rate of other antibodies did not increase. These results illustrate that changes in demographics may have been short-lived post-COVID-19.

## 1. Introduction

Classically, the peak age of diagnosis of type 1 diabetes mellitus (T1DM) occurs during the elementary school years and early adolescence [1, 2]. Since the onset of COVID-19 in March 2020, anecdotally many pediatric endocrinologists have been making the assumption that there has been a shift in age of diagnosis of new-onset T1DM to younger children, but not much has been published to support this claim. The incidence of T1DM diagnosis has been on the rise for many years [2, 3], but there have been several papers published showing an even further increase in the overall incidence of new-onset T1DM since COVID-19 began [4–7]. This will be the first large North American study to assess whether the age at diagnosis has changed during the subsequent 3 years within the pediatric population.

This is significant because age at diagnosis can play a role in long-term prognosis of complications, such as diabetic nephropathy and cardiovascular health [8, 9]. Furthermore, younger children (less than age 5, and especially less than age 2) are harder to diagnose due to symptoms being less apparent and usually present with more severe symptoms (i.e., higher rate of diabetic ketoacidosis [DKA]), and are more difficult to treat due to higher insulin sensitivity [3, 10, 11].

Since the onset of the pandemic, multiple studies have demonstrated an increase in the rate of DKA [4, 6, 7, 12–14]. However, the cause of this increase has not yet been determined. Younger age and acute viral infection have been associated with an increased risk of DKA at presentation [15, 16].

Autoimmunity is rare in infants and young children [17]. T1DM is an autoimmune condition that may be more common in children with a genetic predisposition, but it also requires a trigger to initiate. Viruses are a common trigger for T1DM and autoimmunity, and COVID-19 may be a strong trigger [18]. Notably, the ACE-2 receptor expressed strongly on pancreatic beta-cells is a binding site for the COVID-19 virus, which may be a specific trigger for T1DM related autoimmunity [3, 4, 19].

The primary purpose of this study was to assess whether the average age at diagnosis of T1DM has shifted since the onset of the COVID-19 pandemic, and to determine whether any observed changes are transient or have persisted over time. The secondary purpose of this study was to evaluate whether there was a change in other factors when compared with prior to the pandemic such as rate of DKA at diagnosis, hemoglobin A1c (HbA1c) at diagnosis, and rates of other autoimmune markers at diagnosis, such as anti-thyroperoxidase (TPO) or anti-tissue transglutaminase (TTG)-IgA antibodies, and whether these changes are enduring or reverting to the previous mean.

## 2. Methods

An Institutional Review Board (IRB)-approved retrospective chart review was completed using electronic medical records. Eligible participants were determined from a pre-existing data set of all patients diagnosed with diabetes mellitus and seen at a large academic children's hospital. Patient data extracted from charts included age, date of diagnosis, sex, race, and relevant laboratory studies at diagnosis. The inclusion criteria were age 18 years or younger with a diagnosis of new-onset T1DM initially seen at Akron Children's Hospital between February 2017 and December 2022. Patients who transferred care to Akron Children's Hospital after their initial diagnosis, were diagnosed outside the stated dates, were older than 18 years of age at diagnosis, or were diagnosed with another type of diabetes (type 2 diabetes mellitus, cystic fibrosis-related diabetes, or medication-induced diabetes) were excluded from the study. The before COVID era was determined from February 2017 to February 2020. The during COVID era for the time, following the emergence of COVID-19, was defined as March 2020 through December 2022. Age groups determined by age at diagnosis were defined as <2, 2–5, 6–12, and 13–18 years of age. Patients were considered to have DKA if they presented with hyperglycemia (glucose >200 mcg/dL), acidosis (pH <7.3 and/or bicarbonate level ≤15 mmol/L), and ketosis (presence of urine ketones on urinalysis or β-hydroxybutyrate >3 mmol/L). A patient with celiac disease was defined as a positive TTG-IgA antibody and having the diagnosis made after seeing a gastroenterologist (either clinically or after an esophagogastroduodenoscopy).

Data analysis was performed using SAS (version 9.4; SAS Institute, Inc., Cary, NC, USA). Chi-squared test was used to assess for differences in patient demographics (sex and race) as well as diagnosis of DKA at presentation, presence of TPO antibodies at diagnosis, elevation in TTG-IgA levels at diagnosis, rate of diagnosis by age group, diagnosis of celiac disease, and Hashimoto's thyroiditis requiring treatment with levothyroxine between the two time periods. Student's *t*-test and ANOVA were used to determine differences by time period and year, respectively, regarding age at diagnosis and HbA1c at diagnosis. Student's *t*-test was used to determine differences in HbA1c levels and age at diagnosis between those who presented in DKA and those who did not. Comparisons between years and between age groups were obtained by multiple comparisons using Bonferroni correction (alpha). Statistical significance was set at *p*  < 0.05.

## 3. Results

**Figure 1:**
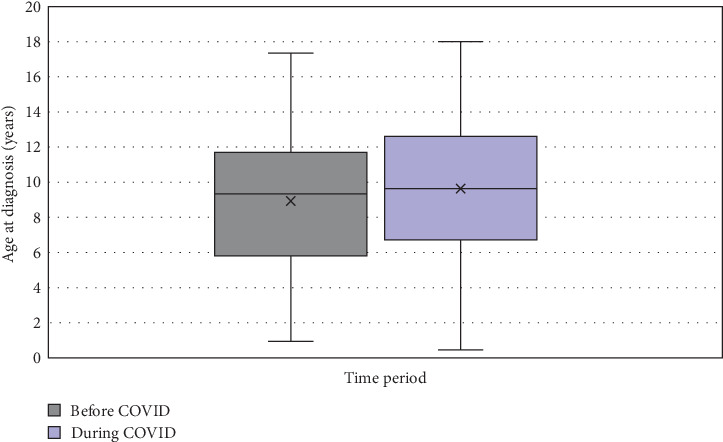
Average age of diagnosis by time period (before to during COVID).

**Figure 2:**
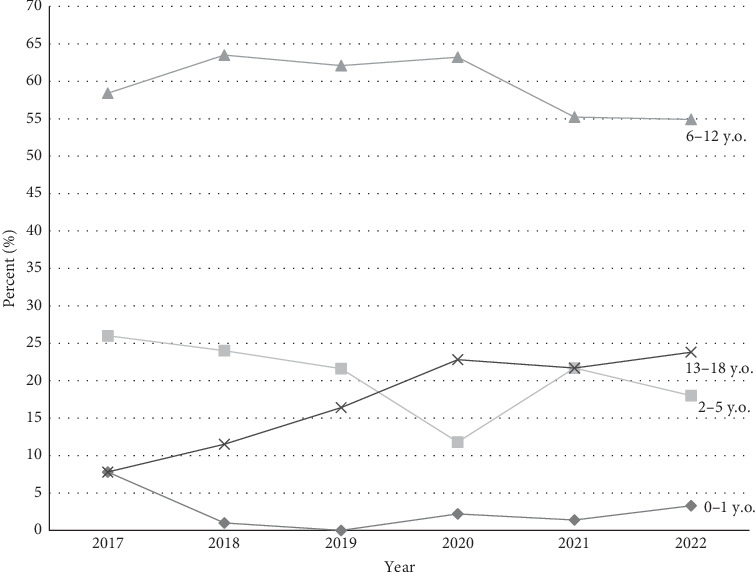
Age distribution at diagnosis by year.

Six hundred and ninety-eight patients met the inclusion criteria; 320 patients in the before COVID era and 378 patients in the during COVID era with demographics and analysis summarized in Table 1. No significant differences were noted between the time periods regarding sex assigned at birth or racial identity. Mean age of diagnosis increased from 8.9 years in the before COVID era to 9.6 years of age in the during COVID era with a mean difference of 0.7 years (CI, 0.08–1.25; *p*=0.025), seen in Figure 1.  Figure 2 shows that there was a significant difference in the mean age of diagnosis each year (*p*=0.0036) and a difference in the distribution of diagnosis in each age group (*p*=0.0021). Additionally, there was a significant change in distribution of age groups from the before COVID era to the during COVID era (*p*=0.0065), specifically between the 13–18 age group compared to the 2–5 age group (*p*=0.012) and 6–12 age group (*p*=0.043). There was a significant decrease in rate of new cases among the 2–5 age group from 26% of cases in in 2017 to 12% of cases in 2020 (*p*=0.0011), subsequently increasing to 18% of cases in 2022. New cases in the 13–18 age group were steadily increasing prior from 7.8% of cases in 2017 with subsequent increases until 21.7% in 2020, with no significant change in proportion of cases through 2022.

### 3.1. DKA and HbA1c

**Figure 3:**
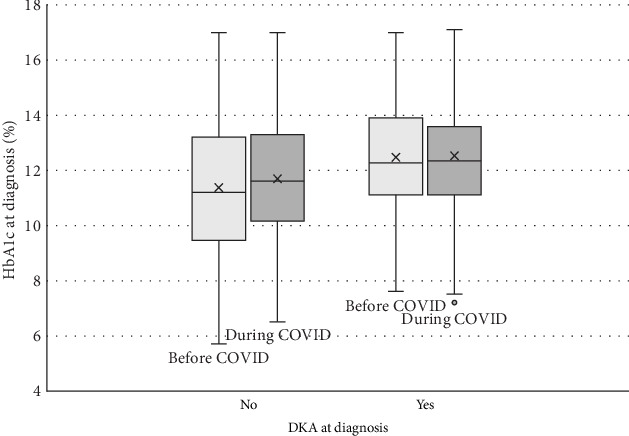
HbA1c at diagnosis, comparing before to during COVID and DKA status.

The correlations between time periods with HbA1c and DKA are summarized in Table 2. The associations between individual characteristics and DKA at diagnosis are summarized in Table 3. Patients were 1.6 times more likely to present in DKA at initial presentation during the during COVID era than in the before COVID era (*p*=0.003; OR, 1.6; CI, 1.2–2.1). Patients who presented in DKA had higher HbA1c levels at diagnosis (12.5%) than patients who did not present in DKA (11.5%; *p* < 0.001). However, there was no significant change in average HbA1c between the before COVID and during COVID eras, even when only looking at those in DKA (Figure 3). Additionally, while there was no difference in average age in those with DKA vs. not in DKA, there was a significant difference in the rate of DKA by age group (*p*=0.0046), with a much higher percentage of those 0–1 years presenting in DKA than the older age groups.

### 3.2. Antibodies

The correlations between TTG-IgA antibodies and TPO antibodies with other demographic factors are summarized in Tables 2 and 4. There were no significant changes in the proportion of patients testing positive for TPO antibodies by year or between the two time periods. There was a significant *decrease* (*p*=0.018) in patients with elevated TTG-IgA from the before COVID era (12.5%) to during COVID (7.2%). There were no significant differences in antibody positivity rate by age or sex.

## 4. Discussion

Contrary to anecdotal belief, our study found that the average age at diagnosis of T1DM increased during the pandemic. To our knowledge, this is the largest study evaluating before and during COVID demographic changes in pediatric patients diagnosed with T1DM. Smaller studies with a few hundred patients did not demonstrate any change in age at diagnosis [12, 20]. Another study noted an increase in the proportion of patients under the age of 4 years being diagnosed, but excluded patients over the age of 14 years (which they note are not seen as much at pediatric institutions in Spain) and only reported on cases through March 2021 [21]. In our study, there was a significant decrease in the diagnosis of patients ages 2–5 years in 2020, but this was followed by normalization of new cases among this group in the more recent years, which may have led to our anecdotal belief that more toddler-aged children were being diagnosed in the during COVID era. This may have been due to delay in presentation to health care while families were stuck home in quarantine or lack of recognition of symptoms by daycares and schools, as well as decreased exposure to any seasonal virus. These effects may vary across countries due to differences in isolation measures.

The contrast in these results illustrate that changes in demographics and distribution may have been short-lived after the start of COVID-19 and may be returning to before COVID distribution (possibly due to strict quarantine lessening during 2021–2022). Further studies over the upcoming years will be needed to elucidate whether any age-related demographic changes will persist. Other demographics, specifically sex and race, remained unchanged, suggesting that this change in the average age at diagnosis was not related to any other demographic factors. While this is a single-center study, our children's hospital provides care to a large catchment area of over 20 counties in multiple states, with a broad range of socioeconomic status. Additionally, it will be pertinent to monitor for any unique changes in morbidity and mortality, such as changes in glycemic control, cardiovascular disease, and diabetic nephropathy [8, 9, 22] given the relationship with age of diagnosis.

Initially, we hypothesized that the overall incidence of diabetes increasing was a temporary change secondary to the virus acting as a trigger in patients who were predisposed to develop T1DM at a later age (meaning these patients were likely to eventually be diagnosed, but COVID acted as a trigger to a large population all at once and they were diagnosed younger than they would have been otherwise). Our finding of an increase in the average age of diagnosis contradicts this theory. Furthermore, we noted a decrease in the number of patients testing positive for TTG-IgA antibodies at diagnosis and no change in TPO antibody positivity at diagnosis. These findings suggest that demographic changes during COVID-19 were not necessarily due to an increase in autoimmunity. There was no change in our testing processes from before to during COVID, so these findings are unique and unexpected, and should be an area of future study. Population-level studies have not shown an increased rate of antibody-negative T1DM, supporting an autoimmune mechanism behind the increased incidence of T1DM since the onset of the COVID-19 pandemic [23]. This further supports the theory that the COVID-19 virus causes both direct and indirect destruction of pancreatic beta cells, likely related to the binding of the virus to the ACE-2 receptor on pancreatic beta cells as seen in previous in vitro cell studies [3, 4, 19]. Interestingly, while autoimmunity is typically female predominant, T1DM does not show a specific sex predominance [24, 25]. Further studies in upcoming years can help to further elucidate the relationship between COVID-19, its direct effect on beta cells, and the induction of autoimmune disease in susceptible populations.

Consistent with prior studies, we noted an increase in the rate of DKA at initial diagnosis in the during COVID-19 era. Some theorize that children are simply becoming sicker faster, with COVID-19 acting as a trigger for autoimmune damage to the pancreatic beta cells or related to the angiotensin converting enzyme receptor 2, the binding site for the COVID-19 virus, being strongly expressed on pancreatic endocrine cells [3, 4, 19, 21]. Others theorized a delay in care due to the pandemic [7, 12]; however, one retrospective study did not find any difference in time from symptom onset to diagnosis during the pandemic era [10]. Our findings do not support either theory, as average HbA1c in those presenting in DKA remained unchanged, suggesting that the time from onset to presentation in DKA was not prolonged. This highlights the need for additional studies on the mechanism connecting COVID-19 and DKA. We also observed that the rate of DKA is higher among younger patients, aligning with findings from other studies [15, 16]. This may be attributed to the challenges in recognizing symptoms in infants and toddlers, underscoring the importance of understanding shifts in patient demographics.

Strengths of this study include the largest evaluation of age and other demographic data thus far, comparing patients diagnosed with T1DM before and after the emergence of the COVID-19 virus. The results of this study should be interpreted within the context of several limitations. The patient population consisted of patients treated at a single children's hospital and may not be generalizable to the rest of the T1DM population. Additionally, we did not examine whether individual patients were diagnosed with COVID-19 prior to diagnosis of T1DM, and therefore were unable to assess for a direct causal relationship between the virus and diagnosis. The direct clinical relationship between COVID-19 and diabetes antibody positivity or beta-cell destruction may be an area of future study. We also did not examine whether there was a change in rate of diabetes antibody positivity from pre- to post-COVID, however this may be an area for future study.

## 5. Conclusion

In conclusion, our study showed a significant increase in the age of diagnosis in the during COVID era, which contradicted our hypothesis that there was an increase in younger patients being diagnosed. Further studies are needed to investigate the mechanism leading to an increase in DKA and T1DM in the during COVID-19 era, as well as the effect on long-term care for and management of T1DM with regard to age of diagnosis and subsequent outcomes. Understanding the changing demographics of this population is also paramount to ensure timely recognition of new onset diabetes and DKA. As new therapies emerge that can delay the onset or progression of T1DM, proactive surveillance and early detection will become increasingly essential.

## Figures and Tables

**Table 1:** Demographics and clinical characteristics by time period.

Demographics		Time period		*p*-Value
		Before COVID, *n* = 320	During COVID, *n* = 378	
Age at diagnosis (years)	Mean (SD)	8.9 (3.8)	9.6 (4.0)	0.025*⁣*^*∗*^
	Median (min–max)	9.3 (0.9–17.3)	9.6 (0.4–18.0)	

Age group, *n* (%)	0–1	9 (2.8)	7 (1.9)	0.0065*⁣*^*∗*^
	2–5	73 (22.8)	66 (17.5)	
	6–12	196 (61.3)	219 (57.9)	
	13–18	42 (13.1)	86 (22.8)	

Sex, *n* (%)	Female	157 (49.1)	170 (45.0)	0.2807
	Male	163 (50.9)	208 (55.0)	

Race group, *n* (%)	African American/black	23 (7.2)	42 (11.1)	0.2423
	Asian/native Hawaiian and other Pacific islander	1 (0.3)	3 (0.8)	
	Unknown	5 (1.6)	6 (1.6)	
	White or Caucasian	280 (87.5)	320 (84.7)	
	More than one race	11 (3.4)	7 (1.9)	

*⁣* ^*∗*^Significant value (*p* < 0.05).

**Table 2:** Clinical characteristics and comorbidities by time period of diagnosis.

Clinical characteristics	Time period		*p*-Value	OR (95% CI)
	Before COVID, *n* = 320	During COVID, *n* = 378		
DKA at diagnosis, *n* (%)			0.003	1.6 (1.2, 2.1)*⁣*^*∗*^
No	190 (59.4)	182 (48.1)	—	—
Yes	130 (40.6)	196 (51.9)	—	—
A1c at diagnosis			—	—
Mean (SD)	11.8 (2.5)	12.1 (2.2)	0.0858	—
Median (min–max)	11.7 (5.7–17.0)	12.1 (6.5–17.1)	—	—
Celiac disease, *n* (%)			0.873	—
No	21 (53.9)	14 (51.9)	—	—
Yes	18 (46.20	13 (48.2)	—	—
Positive TPO antibodies, *n* (%)			0.158	—
No	253 (83.8)	318 (87.6)	—	—
Yes	49 (16.2)	45 (12.4)	—	—
Elevated TTG at diagnosis, *n* (%)			0.018	0.54 (0.33, 0.91)*⁣*^*∗*^
No	280 (87.5)	348 (92.8)	—	—
Yes	40 (12.5)	27 (7.2)	—	—
Hashimoto's diagnosis, *n* (%)			0.085	—
No	34 (69.4)	38 (84.4)	—	—
Yes	15 (30.6)	7 (15.6)	—	—

*⁣* ^*∗*^Significant value (*p* < 0.05).

**Table 3:** Characteristics by DKA status at diagnosis.

Characteristics	DKA at diagnosis		*p*-Value	Mean difference (95% CI)
	No (*n* = 372)	Yes (*n* = 326)		
A1c				
mean (SD)	11.5 (2.5)	12.5 (2.0)	<0.0001*⁣*^*∗*^	−0.97 (−1.3, −0.6)
median (min–max)	11.4 (5.7–17.0)	12.3 (7.2–17.1)	—	—
Sex, *n* (%)			0.729	
Female	172 (46.2)	155 (47.5)	—	—
Male	200 (53.8)	171 (52.5)	—	—
Age at diagnosis				
Mean (SD)	9.4 (3.9)	9.1 (4.0)	0.338	—
Median (min–max)	9.4 (1.7–18.0)	9.6 (0.4–17.8)	—	—
Age group, *n* (%)			0.0046*⁣*^*∗*^	
0–1	2 (0.5)	14 (4.3)	—	—
2–5	80 (21.5)	59 (18.1)	—	—
6–12	216 (58.1)	199 (61.0)	—	—
13–18	74 (20.0)	54 (16.6)	—	—

*⁣* ^*∗*^Significant value (*p* < 0.05).

**Table 4:** TPO and TTG antibody positivity by time period, sex, and age.

Characteristic	TPO antibodies		*p*-Value	TTG at Dx		*p*-Value
	No	Yes		No	Yes	
Time period			0.158			0.018*⁣*^*∗*^
Before COVID	253 (44.3)	49 (52.1)	—	280 (44.6)	40 (59.7)	—
During COVID	318 (55.7)	45 (47.9)	—	348 (55.4)	27 (40.3)	—
Gender			0.261			0.649
Female	262 (45.9)	49 (52.1)	—	291 (46.3)	33 (49.3)	—
Male	309 (54.1)	45 (47.9)	—	337 (53.7)	34 (50.8)	—
Age at diagnosis			0.598			0.285
Mean (SD)	9.2 (3.9)	9.4 (3.6)	—	9.3 (3.9)	8.8 (3.6)	—
Age group, *n* (%)			0.264			0.274
0–1	15 (2.6)	1 (1.1)	—	16 (2.6)	0	—
2–5	117 (20.5)	13 (13.8)	—	121 (19.3)	17 (25.4)	—
6–12	334 (58.5)	64 (68.1)	—	372 (59.2)	41 (61.2)	—
13–18	105 (18.4)	16 (17.0)	—	119 (19.0)	9 (13.4)	—

*⁣* ^*∗*^Significant value (*p* < 0.05).

## Data Availability

The data that support the findings of this study are available upon reasonable request from the corresponding author (RH). The data are not publicly available due to the presence of Protected Health Information (PHI), which could compromise the privacy of research participants.
